# Empyema

**Published:** 1918-11

**Authors:** 


**Empyema**. By Hugh McKenna (Chicago) Camp Pike, Little Rock, Ark. *Journal A. M. A.*, Aug. 31, 1918.

The author reports the results of a study of 155 cases of empyema resulting as a sequel of the 1,300 cases of pneumonia treated at the Camp Pike base hospital. All the medical officers from the beginning were instructed to examine pneumonia patients carefully so as to determine the presence of pus in the pleural cavity. On the admission of these patients to the surgical service, after the condition was determined by roentgenography, blood cultures were taken. The roentgenographs and examinations were repeatedly made, and the aspirated fluids examined in the laboratory. The bacteriologic findings in the pleural fluid showed S. haemolyticus in 101 cases and the pneumococcus in twenty-five; Type I was found in two cases, Type II in seven, and Type IV in six. Seven cases were not agglutinated. At the beginning the operation of costectomy or thoracotomy was used as practically the routine method, though in a few cases aspiration was carried out and 2 per cent. formaldehyd solution in glycerin was injected into pleural cavity. In the drainage cases, as a rule, Brewer's tubes were used. In some of these cases negative pressure was produced by means of Woulfe's bottles. The separate pus pockets observed in many cases are of interest, and in order to drain these cavities an operation was devised in which 3 1/2 inches of the fourth rib were resected and retraction was produced so that the gloved finger might sweep over the anterior border of the lung into this space, which allowed pus pockets to be reached anywhere between the diaphragm and the manubrium sterni. Observation of these cases gave a number of reasons for not opening the pleural cavity, and the following plan was decided on : To investigate the disease very carefully and extensively by a team formed of members who were named from the surgical, roentgenologic, medical, laboratory, and bacteriologic assistants, to which was added a specially trained nurse. Twenty-five patients have been operated on since April 1st, with one death, and the prognosis for the others is excellent. One point in the operation is specially mentioned, namely, the drainage of the pleural cavity, irrespective of the character of the pus, by means of a Number 14 French rubber catheter, introduced by means of a trocart and cannula just large enough to thread the catheter into the cavity. The catheter is then connected with a 100 cc. glass syringe, and aspiration is intelligently and carefully carried out. If the pus is too thick for aspiration, a small amount of neutral solution of chlorinated soda (Dakin's solution) is allowed to run in. This solution quickly liquefies the pus, so that by repetition of this procedure the entire cavity is emptied. The cannula is withdrawn, leaving the catheter in place, and one-half the number of cubic centimeters of Dakin's solution are allowed to run into and remain in the pleural cavity as correspond to the quantity of pus aspirated during the operation. The process of aspirating through this catheter, and allowing the Dakin's solution to run in, is repeated by a trained ward surgeon three times a day, and two times during the night by an especially trained nurse. When, on examination by the roentgen ray or by aspiration, separate pockets were found, the same procedure was followed. The author regards this operation as having a number of advantages. It is a distinctly minor operation. It minimizes the chance of contamination of the pleural cavity from outside, permits frequent treatments, is not liable to form distressing sinuses, the pus from dependent cavities can be completely evacuated, solidified pus cannot form with the Dakin's solution injection, and danger of injury to the lung is lessened.

